# Disinfection of dental root canals by cold atmospheric plasma: a systematic review and meta-analysis of dental biofilm

**DOI:** 10.3389/froh.2024.1483078

**Published:** 2024-12-03

**Authors:** Lorenzo Sanesi, Valentina Puca, Vito Carlo Alberto Caponio, Morena Pinti, Giuseppe Balice, Beatrice Femminella, Michele Paolantonio, Ilaria Cela, Nagendra Kumar Kaushik, Eun Ha Choi, Rossella Grande, Eloisa Sardella, Vittoria Perrotti

**Affiliations:** ^1^ Department of Clinical and Experimental Medicine, University of Foggia, Foggia, Italy; ^2^ Department of Pharmacy, University “G. d’Annunzio” Chieti-Pescara, Chieti, Italy; ^3^ Department of Innovative Technologies in Medicine & Dentistry, University of Chieti-Pescara, Chieti, Italy; ^4^ Center for Advanced Studies and Technology (CAST), “G. d’Annunzio” University of Chieti-Pescara, Chieti, Italy; ^5^ Plasma Bioscience Research Center, Department of Electrical and Biological Physics, Kwangwoon University, Seoul, Republic of Korea; ^6^ CNR- Istituto di Nanotecnologia (CNR-NANOTEC) UoS Bari, c/o Dipartimento di Chimica, Università Degli Studi di Bari Aldo Moro, Bari, Italy; ^7^ UdA-TechLab, Research Center, University “G. d’Annunzio” of Chieti-Pescara, Chieti, Italy

**Keywords:** biofilm, cold atmospheric plasma, oral microorganisms, plasma medicine, disinfection, dental root canals

## Abstract

**Aim:**

The intricate structure of the tooth root canals has a role in the colonization and biofilm formation in hidden areas that are hardly reached by standard endodontic treatments. This review aims at summarizing data from *in vitro* and *ex vivo* studies for a better understanding of the application of cold atmospheric plasma (CAP) for the disinfection of dental root canals.

**Methods:**

PubMed, Scopus, and Web of Science databases were screened. Characteristics of the included studies were extracted, and a meta-analysis on *ex vivo* studies was carried out to evaluate the effect of CAP on colony forming unit assay of *Enterococcus faecalis (E. faecalis)*. The study was performed following the PRISMA 2020 guidelines.

**Results:**

A total of 31 studies fulfilled the selection criteria. Only 2 investigations reported an indirect plasma treatment, 28 trials used direct CAP administration, while 1 study applied both methods. Most of the studies were conducted on *E. faecalis* using as carrier gas Helium or Argon alone or in combination with Oxygen as well air. A considerable heterogeneity among studies was found regarding treatments which varied for source type, settings, and protocols of application. Despite this, CAP showed effectiveness in reducing *E. faecalis* colony forming unit with a standardized mean difference of 4.51, 95% C.I. = 2.55–6.48, *p*-value < 0.001.

**Conclusion:**

The data demonstrated the antimicrobial effect of direct CAP application against microorganisms. *In-vitro* studies showed an effect that depended on the time and distance of treatment, while the meta-analysis performed on *ex vivo* studies showed that the effect of CAP was independent of time and distance.

**Systematic Review Registration:**

https://doi.org/10.17605/OSF.IO/BJ59V, identifier OSF registries.

## 1 Introduction

Dental pulp and periapical infections are due to the bacterial colonization of the root canals; therefore, primary objective of endodontic treatment is to kill/remove bacteria (1, 2). In the clinical practice, different treatments such as mechanical debridement, chemical irrigation, and ultrasound activation are used (3–6). The complexity of the root canals system makes access to some areas through mechanical instruments almost impossible during endodontic practices. The chemical irrigation on biofilm debridement is inevitable and almost the only solution for cleaning these locations (7). Sodium hypochlorite (NaOCl) is the most employed root canal irrigant to dissolve the pulp tissue, while displaying a wide spectrum of action on bacteria, viruses, and spores (8).

In general, an effective endodontic decontamination protocol provides an 85%–94% success rate (9), decreasing to 65.5%−87.5% in the case of re-treatment (10–14). Many factors are associated to endodontic treatment failure, such as root canal overfilling, root fractures, broken instruments, mechanical perforation, missed or unfilled canals. However, the main cause is the inadequate instrumentation and decontamination of the root canal system with a lack of apical seal, leading to the persistence of intra or extra-radicular bacteria (15–18), in particular *Aggregatibacter actinomycetemcomitans*, *Propionibacterium propionicum,* and *Enterococcus faecalis* (19). Therefore, to overcome the limits of the above-mentioned treatments, especially in demanding clinical conditions such as narrow and tortuous root canals, more effective decontamination methods are required.

Currently, an innovative research field based on the use of Cold Atmospheric Plasma (CAP) has shown a great potential for root canal disinfection (20). Plasma is generated by partial ionization of a gas by supplying enough energy. Interaction between plasma and oxygen and/or nitrogen leads to the formation of reactive chemical species, with antibacterial, antifungal and antiviral properties (21). The main chemical reactive oxygen and nitrogen species (RONS) include hydrogen peroxide (H_2_O_2_), ozone (O_3_), hydroxyl radicals (OH), superoxide (O_2_^−^), singlet oxygen (^1^O_2_), nitric oxide (NO), nitrite (NO_2_^−^), and peroxynitrite (ONOO−), among others (22). The first clinical trial on the therapeutical use of plasma for the treatment of human diseases dates back to 2013 (23). Since then, two types of plasma application modalities have been developed:

(1) Direct treatment involving direct exposure of the biological target (e.g., cells *in vitro*) in the presence of a liquid or not.
(2) Indirect treatment involving the treatment of a liquid (e.g., saline solution, and bacterial culture medium) or hydrogels (plasma treated hydrogels, PTH), and the subsequent application of these plasma treated products onto the biological target, *in vitro* or *in vivo*, allowing treatment of internal and less accessible target sites.

Due to the promising anti-microbial and regenerative effects, CAP may find different applications in dental medicine, such the treatment of periodontal and peri-implant diseases (24, 25), caries (26) and dental bleaching (27), and endodontics (20). However, only few studies evaluated root canal disinfection using CAP-based approaches showing contrasting results and underling the lack of standardization in the protocols (i.e., direct vs. indirect approach, exposure time, feeding gas, plasma sources and treatment distance), which can make unfruitful the efforts towards a clinical translation of CAP application.

Therefore, the present study aimed to systematically review the literature published up to now on the use of CAP for root canal decontamination with the purpose to shed light on the definition of a proper protocol of application to guarantee a successful decontamination of the target site (i.e., limited damage to the surrounding biological tissues and reduced incidence of endodontic failures and reinfections).

## 2 Materials and methods

This systematic review was performed according to the guidelines of the Preferred Reporting Items for Systematic Reviews and Meta-analyses (PRISMA) statement (28). The review was registered on OSF registries https://doi.org/10.17605/OSF.IO/BJ59V.

### 2.1 Eligibility criteria

#### 2.1.1 Inclusion criteria

The search was limited to studies published in English without any year restriction. To be eligible for inclusion, these criteria had to be satisfied:

1. Case-control design of *in vitro* or *ex vivo* studies on dental root canal treatment by CAP.
2. Biological, microbiological, and physic-chemical effects of CAP on dental root canal treatment.

#### 2.1.2 Exclusion criteria

The exclusion criteria were as follows:

1. Proceedings, abstracts;
2. Short communications;
3. Systematic reviews and meta-analyses;
4. Ecologic studies;
5. *in vivo* studies.

### 2.2 Search strategy

An electronic search on PubMed, Scopus, and Web of Science was performed to identify suitable studies, using the following terms and keywords alone or in combination:

(“cold atmospheric plasma” OR “cold plasma” OR “low temperature plasma” OR “kinpen med” OR “non-thermal atmospheric pressure plasma” OR “non thermal atmospheric pressure plasma” OR “cold physical plasma” OR “plasma medicine” OR pam OR cap OR “plasma activated medium” OR “cold atmospheric-pressure plasma” OR “plasma gases” OR “plasma activated liquid” OR “cold argon plasma” OR “plasma jet” OR “air plasma”) AND (biofilm OR biofilms OR “oral biofilm” OR “oral microorganism*” OR “biofilm colonization*” OR “dental plaque” OR “dental deposit*” OR “materia alba” OR “endodontic disease*” OR “dental canal” OR endodontics OR “conservative dentistry” OR “dental root”).

The first search was performed on October 16th, 2022. The last electronic search was performed on January 12th, 2024. In addition to the electronic search, a hand search was undertaken by checking the references of the included studies to identify further eligible studies. A reference manager software program (Mendeley Software Manager) was used, and the duplicates were discarded first electronically, then by checking the resulting list manually. The search strategy, adapted for PubMed, Web of Science, and Scopus is shown in Supplementary Table S1.

### 2.3 Focused PICO question

Based on the methodology of the study, and specifically if *ex vivo* or *in vitro*, the question is formulated:

Focused question: which were the biological, microbiological, and chemical-physical effects of CAP on *in vitro* and *ex vivo* bacterial cultivation of species involved in endodontic pathology?

#### 2.3.1 *In vitro* articles

Participants: monospecies and multispecies biofilms commonly isolated from the endodontic space.

Intervention: direct or indirect CAP treatment.

Comparison: traditional endodontic treatments or different CAP treatment protocols.

Outcomes: microbiological and physic-chemical changes of biofilm.

#### 2.3.2 *Ex vivo* articles

Participants: extracted teeth eligible for endodontic treatment.

Intervention: direct or indirect CAP treatment.

Comparison: traditional endodontic treatment or different CAP treatment protocols.

Outcomes: Disinfection and physicochemical changes of dental root tissues.

### 2.4 Selection of studies

Retrieved citations were independently screened by two authors (LS and GB), and relevant studies were identified based on title and abstract. If those did not provide sufficient information about the inclusion criteria, the full texts were evaluated to assess eligibility. Any disagreement was solved by discussion, and a third reviewer was consulted to make final decisions (VP). This author also calculated a kappa-statistic value to ascertain the level of reviewers’ agreement.

### 2.5 Data extraction and method of analysis

Two reviewers (LS and GB) independently extracted data from all the included studies using a predesigned extraction form (Microsoft Excel 2020, Microsoft Corporation, Redmond, WA, USA) for data collection and descriptive analysis. Extraction sheets were organized to collect:

1. CAP device features with the following items: type of plasma device and developer, CAP settings (i.e., gas flow rate, feeding gas, pulse frequency, pulse, temperature, application distance, application time, total energy, and power).
2. study characteristics: first author and year, study type (i.e.: *ex vivo*, *in vitro*), biofilm cultivation characteristics, sample size, outcomes, and results.

### 2.6 Risk of bias assessment

Two reviewers (LS and GB) evaluated the quality of studies by risk of bias assessment. Diverse scales were employed because of the heterogeneity of study designs of the included trials.

For *in vitro* studies, criteria based on the guidelines for creating bacterial culture studies were modified for the methodological quality of *in vitro* research. Six items were considered: 1. condition of bacterial culture before experimentation; 2. condition of bacterial culture during treatment; 3. description of methodology to evaluate outcomes; 4. case-control description; 5. multiple experiments performed; 6. descriptions of plasma settings and devices. Studies that satisfied at least 5 over 6 of the stated criteria were deemed to have a low risk of bias (Supplementary Table S2).

For *ex vivo* studies, the following criteria were used: 1. randomization of teeth in the different groups; 2. presence or absence of caries or restoration in teeth considered; 3. use of materials according to the manufacturer's instructions; 4. use of teeth with similar dimensions; 5. endodontic treatment performed by the same operator; 6. description of characteristic of bacterial cultivation; 7. blinding of the operator of the testing machine and the accuracy of plasma protocol description (29, 30).

The article received a “✓” (yes), fulfilling the investigated parameter; otherwise, it received a “–” (no) in case of negative response, U (Unclear) if the information was not clear in the article and N/A (not applicable) if it was not possible to obtain the information. Studies were identified as having a low risk of bias if they met at least 5 over 6 of the specified criteria, whereas studies that did not satisfy 2 or more than 3 of the reported elements were categorized as having a medium or high risk of bias (Supplementary Table S3).

### 2.7 Meta-analysis

Meta-analysis was carried out on *ex vivo* studies. Changes in standardized mean difference (SMD) of colony forming unit (CFU) counts were pooled for the meta-analysis of the included studies. Data were input as log (CFU/ml) and standard deviations (SDs) for both test (CAP treatment) and control group. Estimation of overall SMD and relative 95% confidence intervals (95% C.I.) was performed as Hedges’ g weighted data. These results were graphically represented as forest plots. Heterogeneity assessment was inspected to set a random or fixed-effect meta-analysis. Cochran's Q test was used to evaluate study heterogeneity and the I^2^ index was used to measure it. A random model was set for I^2^ values more than 50%, otherwise a fixed effect model was used (31).

To further assess heterogeneity, subgroup analysis was performed for the different feeding gas employed in CAP treatment; relation between effect size and respectively time of exposure treatment, application distance and publication year was inspected with meta-regression.

## 3 Results

### 3.1 Study selection

For this review 3,699 potentially relevant records from the databases were analysed following PRISMA statement. After the removal of duplicates, the articles were screened based on their titles and abstracts, and 3,520 articles were excluded. A total of 31 full-text articles were evaluated, and 2 were subsequently excluded for the reasons listed in Supplementary Table S4. The reference list of included studies was also reviewed to identify any further study missing in the electronic research and 2 articles were selected for inclusion.

**Figure 1:**
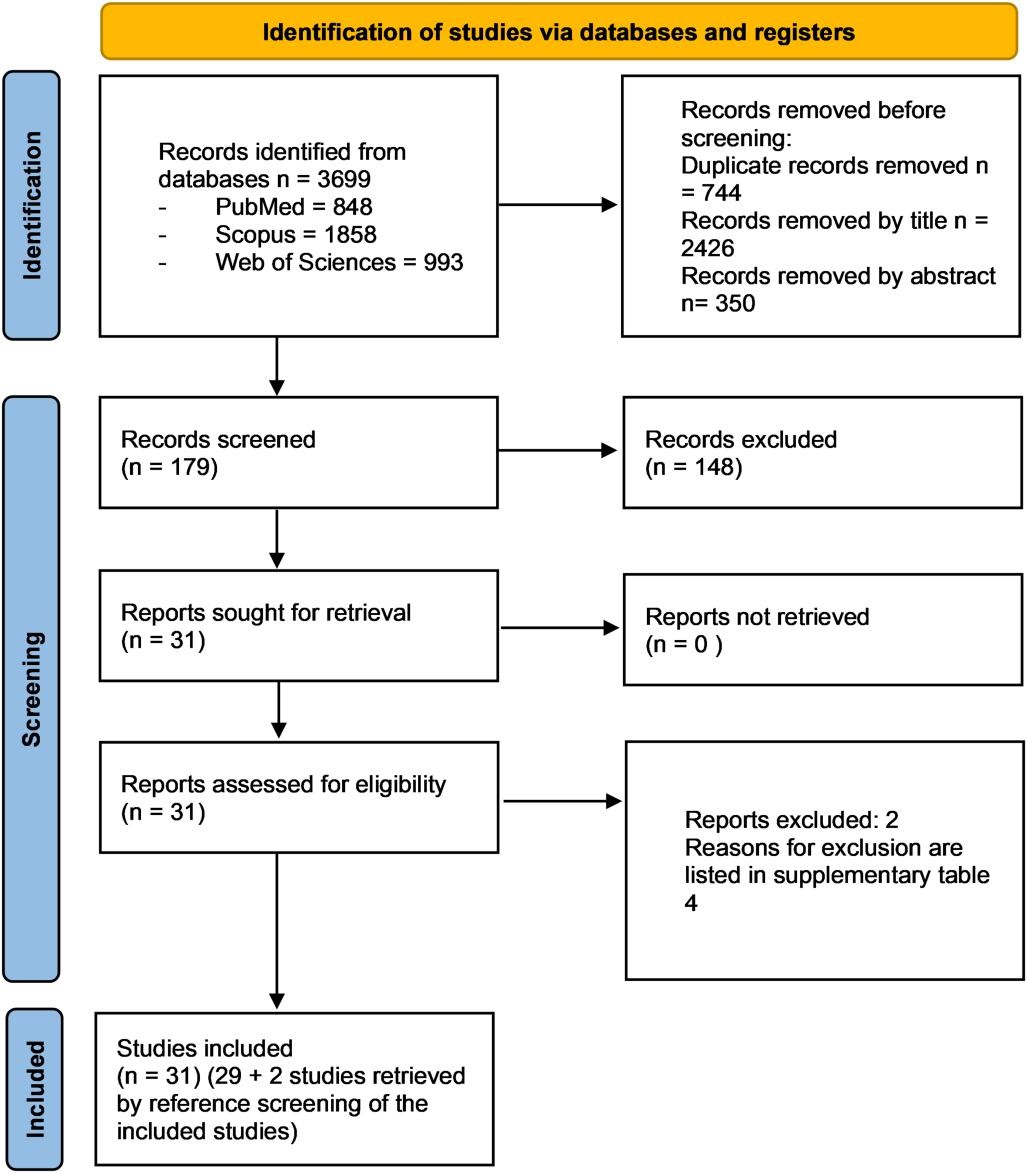
PRISMA 2020 flow diagram of the screening process. In total, 31 studies were included in the present systematic review.

Therefore, a total of 31 studies fulfilled the selection criteria of the present review and were included in qualitative analyses (Figure 1). The value of the kappa-statistic was 0.83 which indicated a moderate agreement between the 2 reviewers, following the formula provided by the Cochrane Handbook for Systematic Reviews of Interventions.

### 3.2 General characteristics of included studies

Based on the study design, the studies were categorized into 2 different groups:

- 15 *in vitro* studies that investigated the antibacterial activity of CAP against *E. faecalis* (32–38), *Candida albicans* (*C. albicans*) (39–43), *Escherichia coli (E. coli)* (44), *Streptococcus mutans (S. mutans)* (36, 45, 46) and *Streptococcus sanguinis (S. sanguinis)* (45). Studies were carried out on biofilms on: agar (33, 35, 40, 42), PMMA disc (39, 43), simulated root canals in plastic blocks (34, 37), on cover glass (32), 96 well plate (41), 48 well plate (45), hydroxyapatite blocks (46), dentin blocks (38) porous sterile cellulose (33). Only 2 studies are carried out in microorganism in suspension (36, 44). Among papers showing the antibacterial properties of CAP, 4 studies were conducted in Germany (35, 37, 40, 43), 3 in China (33, 34, 38) and in Brazil (41, 42), 1 study was performed in Turkey (39), Taiwan (32), Japan (36), Ireland (44), USA (45), and South Korea (46), respectively.
- 16 *ex vivo* studies investigated the decontamination efficacy of CAP in root canals of human-extracted teeth, mainly on *E. faecalis* (1, 47–59), followed by 1 study using a human-derived microbial culture (60), and *C. albicans* (61), respectively. The sample size (teeth) in the articles ranged from 24 to 200, and the articles were published between 2011 and 2023. Among papers on decontamination efficacy on *ex vivo* experiments, five of them were conducted in Germany (48, 49, 60, 61), 6 articles were conducted in China (1, 38, 50, 51, 56, 59), 2 studies in Iran (47, 55), 1 in Mexico (54), 1 in USA (57), 1 in India (58) and 1 in Turkey (52).

### 3.3 Results of risk of bias assessment

Analysing risk of bias in *in vitro* studies, 14 presented a low risk of bias (32, 33, 35–46); while 1 presented a moderate risk (34) (Supplementary Table S2).

Referring to *ex-vivo* studies, none was rated as at a high risk of bias; 5 studies presented a moderate risk of bias (1, 47, 49, 50, 60), while 11 studies showed a low risk of bias (48, 51–59, 61) (Supplementary Table S3).

### 3.4 *in vitro* studies on relevant pathogens

Ten studies applied CFU count to evaluate the biofilm viability (32–36, 40, 41, 43, 44, 46). Two studies used MTT to evaluate viability and biofilm formation (39, 45). Scanning Electron Microscopy (SEM) was used in 5 articles to evaluate the bacterial surface morphologies (32, 33, 38, 39, 46). Delben et al. (42), Zhu et al. (38) and Yoo et al. (46) showed the bacterial viability via confocal laser microscopy (CLSM) analysis. Moreover, Delben et al. (42) evaluated the growth inhibition zone. All results are summarized in Supplementary Table S5.

#### 3.4.1 Biofilm development

Most studies have utilized *E. faecalis* (32, 33, 35, 36, 38) and *C. albicans* (39–43). The reported cultivation times for *E. faecalis* in biofilms are highly variable, ranging from 18 h (36) to 7 days (38). Similarly, the cultivation time for *C. albicans* varies significantly across studies, from 24 h (39, 41, 43) to 16 days (43). *S. mutans* was cultured for 24 h by Hirano et al. (36) and Yoo et al. (46), while Liu et al. (45) employed a 7-day biofilm. Charoux et al. (44) and Liu et al. (45) applied *E. coli* cultured for 72 h and *S. sanguinis* cultured for 7 days, respectively.

#### 3.4.2 Biofilm viability

Thirteen studies (32–36, 38–43, 45, 46) applied a direct plasma treatment, while only 1 study (44) used an indirect approach. Five studies (32, 33, 35, 36, 38) analysed the effect of plasma treatment on *E. faecalis*. All these studies except Zhu et al. (38) found a decrease in CFU increasing with the employment of different treatment times. Chang et al. (32) observed a significant decrease in CFU starting from 2 min, and after 15 min of treatment a complete eradication was observed. Cao et al. (33) and Theinkom et al. (35) observed a significant decrease in CFU starting from 5 min, while Hirano et al. (36) found a significative reductions at 3 min and eradication was observed in half of the time compared to Chang et al. (32) study (15 min). This difference could be determined by a different approach used in the two papers: planar DBD on dry biofilm (32) vs. plasma Jet on bacteria suspensions (36). The plasma jet in the paper of Hirano et al. (36) worked in contact with the biofilm suspension (i.e., direct contact of the ROS/UV and electric field of the plasma with the biofilm) while the planar DBD of the paper of Chang et al. (32) is remotely switched on (diffusion of the plasma produced species toward the biofilm with limited role of electric field and UV-radiation against biofilm). Zhu et al. (38) did not measure CFU but observed a decrease in live/dead microorganism ratio by CLSM analysis following 5 min. treatment with plasma loaded microbubbles (PMBs) and PMBs + ultrasound (US) compared to control group. The two PMBs groups showed no significant differences. Finally, when a bacterial suspension is used the contemporary effect of stable reactive species produced in the liquid could positively contribute to the efficacy of plasma processing.

Five studies (39–43) analysed the effect of plasma on *C. albicans*. Leite et al. (41) showed a decrease in CFU after 5 min of treatment, Maisch et al. (40) and Matthes et al. (43) observed respectively a diminution of CFU after 7 min and after 10 min of treatment. Avukat et al. (39) and Delben et al. (42) reported similar results at 2 min.

Avukat et al. (39) differently from the other paper used a RF sputtering under vacuum thus increasing dramatically the effect of ion bombardment does not present in atmospheric pressure plasmas. Surprisingly Delben et al. (42) obtained the same results using a plasma jet by demonstrating how the growth inhibition zone increases with the proximity of the plasma application and with an increase in exposure time.

*S. mutans* and *S. sanguinis* resulted also sensitive to direct CAP treatment, and a significant reduction in CFU was achieved in treatment time ranging from 1 to 2 min (45, 46). The efficacy was tested for both single- and dual-species biofilms (45). In summary, direct treatment with plasma shows an effective action against endodontic microorganisms with an action comparable to the traditional methods that have been compared. Furthermore, the application time and distance can influence the effectiveness against the microorganism.

The only study on indirect CAP application (plasma-activated water) was performed on *E. coli* and a CFU reduction was observed after 15 min treatment in combination with acoustic ultrasounds (44). The paper of Hirano et al. (36) shows experiments in which the plasma jet was faced to a suspension of bacteria in PBS showing efficacy on biofilm suspension after 7 min of plasma exposure, an approach that compared with that one of Charoux et al. (44) in which plasma jet was faced with distilled water. The major difference between the two approaches lies in the fact that when the plasma interfaces with a suspension the bacteria are affected by both the less stable reactive species (ex. NO, ONOO) and the more stable ones (ex. H_2_O_2_, NO_2_^−^, NO_3_^−^) while in case of an indirect approach like that one proposed by Charoux et al. (44) the bacteria are affected only by long-living species.

#### 3.4.3 Chemical characteristics

Only Chang et al. (32) evaluated reactive species using Optical Emission Spectroscopy (OES) produced by an air based CAP. The result revealed emission lines corresponding to He, neutral O_2_ atom radiation, hydroxyl radicals, and neutral and atomic species of N (32), which might partially explain the anti-microbial activity. Charoux et al. (44) evaluated the concentration of nitrites and H_2_O_2_ produced other than the pH and electrical conductivity of produced plasma activated water (PAW). The pH values of the PAW showed a significant decrease after both cold and thermal plasma treatment. Plasma treatment also led to an increase in nitrate and nitrites, and higher levels of reactive nitrogen species (RNS) are founded in cold PAW than thermal PAW. Moreover, both plasma treatment methods increased the oxidation–reduction potential (ORP) in the treated PAW compared to the control. Finally, the conductivity of PAW generated by the cold plasma jet was higher than thermal plasma jet. Zhu et al. (38) observed an increase of NO and H_2_O_2_ in PMBs group and PMBs + US compared to control group due to the plasma dissolved in solution.

### 3.5 Preclinical models: *in vitro* model in resin

Only 2 studies (34, 37) included in this review used a simulated root-canal model in resin infected by *E. faecalis*.

#### 3.5.1 Biofilm development

Both studies described the contamination protocol. In general, the bacterial suspension of *E. faecalis* was obtained by preliminary growth on agar plates and subsequently, the bacterial solution was inoculated into the simulated root canal. Subsequently, the simulated root canals were incubated aerobically at 37°C for 2 h (37) and 72 h (34).

#### 3.5.2 Biofilm viability

Both studies evaluated antimicrobial effects via the CFU count. Zhou et al. (34) applied plasma jet treatment (He-1%O_2_) for 2, 4, 6, 8, 10 and 12 min. A significant decrease in CFU was observed starting from the 2-minute treatment. Jablonowski et al. (37) compared plasma (Ar) treatment for 3 min with chlorhexidine digluconate (CHX) and sodium hypochlorite (NaOCL). All three treatments showed a significant decrease in CFU compared to the untreated control.

#### 3.5.3 Cell morphology

SEM was used to examine the morphological changes of bacteria located on the simulated root canal wall in the study by Jablonowski et al. (37), while Zhou et al. (34) applied transmission electron microscopy (TEM) and they observed also nuclear chromatin condensation and cell-division arrest.

### 3.6 Preclinical model: *ex vivo* humans

Four-teen studies (1, 47–59) evaluated the CAP effect on *E. faecalis* in *ex vivo* models of extracted root canals. Only 1 study (61) used a strain of *C. albicans.* In 1 study biofilm was not specified (60). Most studies employed direct CAP treatment compared to conventional therapies, such as NaOCl (42, 48, 49, 52, 57, 58, 60, 61), CHX (50, 53, 56, 61), photodynamic therapy (PDT) (47, 48), gaseous ozone delivery system with 2.5% NaOCl (52) and Ca(OH)_2_ (1, 50, 51, 55).

Different assays were performed to assess the biological and chemical reactions. Most articles (1, 47–50, 52–61) investigated the efficacy of CAP by CFU count and live/dead staining, and variations in the bacteria morphology via SEM and CLSM analysis. Reactive species generated by plasma were identified by OES for the plasma phase, and Electron Spin Resonance (ESR) spectroscopy in distilled water (50, 51). All the results are summarized in Supplementary Table S6.

#### 3.6.1 Biofilm development

All the studies (1, 47–53, 60, 61) described the contamination protocol. In general, the bacterial suspension was obtained by a preliminary growth on agar plates and subsequently in brain-heart infusion broth (BHI). Subsequently, the sterilized tooth was immersed in a tube with 2 ml of the bacterial suspension. The tooth is incubated to allow biofilm formation under aerobic conditions and at 37°C. During the incubation period the culture medium was replaced every 2 days. Only 1 study (61) used *C. albicans* to infect root canals; in particular, the operator inoculated fungal suspension in the canals and incubated them aerobically for 7 days at 37°C.

#### 3.6.2 Biofilm viability

Most of the studies (1, 47–50, 52–61), the efficacy of CAP treatment was evaluated by CFU counting. Live/dead staining and CLSM observation were also carried out in 4 studies (1, 50, 53, 60). In general, plasma treatment has been confirmed as a valid option compared to conventional disinfection treatments, such as NaOCl, CHX, Octenidine (OCT), or PDT. A common observation regarding the efficacy of plasma resulted the time-dependent disinfecting ability. The plasma treatment time range varied from 2 min to 15 min, moreover, longer exposure time led to a greater reduction of CFU (1, 47, 49–51, 56). Wang et al. (51) evaluates the rate of bacterial in the root canal after treatment with Plasma microjet (PMJ). Reinfection decreases with the increase of PMJ treatment time. After 40 min, the samples showed no reinfection.

Concerning the fed gas, in Armand et al. (47), the He + 0.5% O_2_ plasma treatment group showed better results in reducing CFU compared to the He plasma alone, especially at 8 min of treatment. Other papers use He/O_2_ or Ar/O_2_ mixtures as gas feed with variable percentage of O_2_ up to 20%. The observed results in these papers suggest a role of O_2_ in the microbial activity of plasma. In few articles (49, 53, 61), in addition to the normal comparison of plasma monotherapy vs. conventional therapies, the effect of possible combination therapies was tested. Specifically, in Kerlikowski et al. (61) a higher disinfecting power was observed when combining O_2_ based CAP with NaOCl. In contrast, Ballout et al. (48) showed that traditional therapies, such as NaOCl, had better disinfecting capacity than plasma treatment. This could be determined by a shorter plasma treatment time compared to the other studies. Though, Schaudinn et al. (60) instead observed how both NaOCl and plasma treatment were effective for biofilm removal although NaOCl was more effective during the same exposure time. Similarly, Arguello-Sanchez et al. (54) and Kumar et al. (58) showed a decrease of CFU starting from 1 to 2 min with NaOCl treatment respectively, while to obtain a similar reduction with CAP treatment it is necessary to treat root canals for at least 5 min (58). Moreover, Arguello-Sanchez et al. (54) treated *E. faecalis* samples for 5 min. using PAW and a direct plasma jet. The authors observed a significant decrease in CFU following direct plasma jet treatment, whereas no such reduction was observed with PAW treatment.

#### 3.6.3 Cell morphology

SEM analysis was used in 7 studies on *E. faecalis* (1, 47–51, 53) and 1 on *C. albicans* (61). A change in the morphology of bacteria was observed. Specifically, a reduced cell size, ruptures, and damaged structures of *E. faecalis* biofilm were observed in the treated samples compared to the control group. In particular Wen et al. (53) observed how the integrity of the bacterial outer membrane was severely damaged and the normal spherical structure dissociated.

#### 3.6.4 Mechanical safety evaluation

Only one study (50) evaluated the values of pulpal dentin microhardness (HV) and roughness (Ra). In this study, plasma treatment was able to remove *E. faecalis* biofilm, without affecting pulpal dentin HV and Ra after different treatment times (3, 6, 9, and 12 min), indicating that CAP did not change the mechanical properties of pulpal dentin.

#### 3.6.5 Chemical characteristics

The development of ROS and RNS species after treatment, was demonstrated via two different assays, ESR and OES, in 1 (51) and 3 studies (47, 50, 51), respectively. Armand et al. (47) analysis observed the presence of reactive O_2_ radicals that oxidized the outer membrane of the microorganism. They observe excited O lines at 285 and 777 nm, OH line at 309 nm, O^+^ line at 427 nm. Li et al. (50) and Wang et al. (51) observed that Ar emission was dominant because Ar is the major component (98%) of the working gas, but also identified a strong atomic O_2_ emission at 777 nm and at 844 nm. O_2_ emission at 777 nm is determined by dissociative excitation by electron collisions from the ground state O_2_ molecules, while O_2_ emission at 844 nm, on the other hand, is determined mainly by direct electron impact with ground state O_2_ atoms.

### 3.7 Types of CAP devices and CAP treatment parameters

All *in vitro* studies (32, 43, 45, 46) used direct plasma treatment except one (44). CAP treatment was delivered directly on the *in vitro* model by means of different devices. 10 studies used plasma jet (33, 34, 36, 37, 39, 41, 42, 44–46), in 2 studies applied Dielectric Barrier Discharge Plasma (DBD) plasma (32, 43), 2 studies used Surface Micro Discharge Plasma (SMDP) (35, 40), and only 1 study applied PMB (38). Five studies (32, 35, 36, 40, 44) used ambient air to produce plasma, 3 studies (33, 34, 39) employed a mixture of O_2_ and He, in 3 studies Ar was the feeding gas (37, 42, 43), 2 studies used a mixture of Ar and O_2_ (43, 45), and finally 1 study N (46), and 1 study He (41). One study applied plasma activated water treatment (44). This technique employs a plasma technology to modify the properties of water, with consecutive generation of reactive species such as radicals, ions, and other highly reactive molecules with documented antimicrobial activity (44). The plasma application protocol varied greatly among studies. The application distance ranged between 2- and 20-mm. Plasma application time varied from 20 s to 30 min. Most of articles did not report the power either the temperature as experimental variables. The plasma sources used in the plasma jet and DBD studies applied a gas flow that is suitable for root canal treatment, while the studies that used SMDP, did not generate a strong gas flow necessary to thoroughly treat complex dental root canal systems.

All *ex vivo* (1, 47–53, 55–58, 60, 61) studies except one (59) used direct plasma treatment. Only Arguello-Sanchez et al. (54) applied both direct and indirect methods. One study (53) shows a direct approach in which root canals are filled with a liquid before plasma treatment. 16 studies used plasma jet (1, 47–61), and 1 study applied DBD plasma (48). Combination of Ar with 1 or 2% of O_2_ was employed in 6 studies (1, 49–51, 57, 61), 5 studies employed He and O_2_ (47, 52, 55, 56, 60), 4 studies used He (47, 54, 58, 59) and 1 study used Ar (48). Indirect plasma treatment was used in two studies (54, 59). Zhou et al. (59) applied plasma jet fed with He and vapours of H_2_O_2_, while Arguello-Sanchez et al. used PAW (54). The application protocol differed widely among studies, including pulse frequency, pulse, flow rate, plasma temperature, source application distance and time, total emitted energy, and power. The pulse frequency ranged between 0.05 to 100 kHz, and the application distance and time ranged between 1 and 10 mm and 1 to 15 min respectively. The power varied between 0.5 and 30 W, with the most commonly used device, Kinpen, which was typically set to a power of 8 W. All the parameters used in these different protocols are summarized and outlined below (Supplementary Table S7).

#### 3.7.1 Temperature evaluation

Thermal effect is one of the physical factors developed in direct treatment along with UV radiation and electromagnetic fields. A thermal imaging camera was used to measure the working temperature of the plasma device in 2 articles (47, 50). The device used by Armand et al. (47) developed a temperature less than 40°C after 4, 6, and 8 min of plasma exposure, while Li et al. (50) detected mean CAP temperatures near the top of the root canal of 30.1°C and a room temperature of 27.6°C. Other authors reported plasma temperatures. Arguello-Sanchez et al. (54) and Leite et al. (41) reported a working plasma temperature below 40°C. Delben et al. (42) reported a plasma temperature at 3 mm of 30.67 ± 0.58°C and at 10 mm 29.83 ± 0.29°C. Habib et al. reported a temperature of plasma between 30 and 65°C (57). Pan et al. (1) reported a working plasma temperature between 25°C and 31°C. Theinkom et al. (35) reported a plasma temperature of 21°C. Wen et al. in 2022 (53) reported a working plasma temperature between 40°C and 70°C. Jablonowski et al. (37) reported a temperature of 42°C at the tip of the plasma jet. Summarizing the temperature range of gases varying from 21°C reported by Theinkom et al. (35). which use ambient air as gas up to the 65°C reported by Habib et al. which use a mix O_2_ and Ar gas for direct plasma treatment, while for indirect plasma treatment authors used a range of temperature between 40°C reported by Wen et al. (53) and Charoux et al. (44) up to 70°C reported in Wen et al. (53).

### 3.8 Meta-analysis

**Figure 2:**
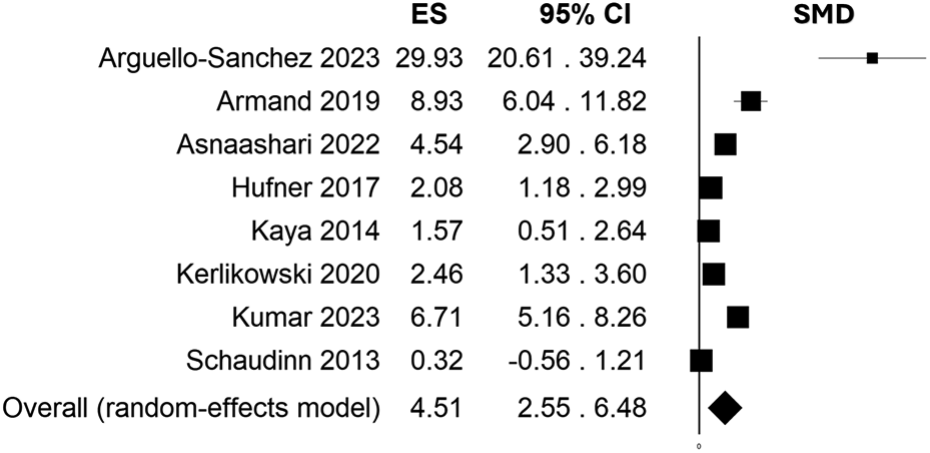
Effect size (ES) estimation by standardized mean difference (SMD) and 95% C.I. showing efficacy of CAP treatment compared to control in reducing overall CFU/ml counts (4.51, 95% C.I. = 2.55-6.48, *p*-value < 0.001).

High heterogeneity emerged as I^2^ resulted 93.82%. For this reason, means and SDs were pooled in a random effects model, and SMD resulted 4.51 (95% C.I. = 2.55–6.48, *p*-value < 0.001) (Figure 2). Heterogeneity I^2^ dropped to 0% when considering only those studies in which treatment was based on Ar/O_2_ while high heterogeneity persisted for He alone and He/O_2_ subgroup. Fixed effects model meta-analysis for Ar/O_2_ subgroup showed a SMD of 2.23 (95% C.I. = 1.52–2.94, *p*-value < 0.001) while random effects model resulted in a SMD of 3.52 (95% C.I. = 0.88–6.16, *p*-value = 0.009) in He/O_2_ subgroup and 17.85 when He alone was employed (95% C.I. = −4.89–40.58, *p*-value = 0.124). Meta-regression showed neither correlation between estimated effect sizes and publication year nor exposure treatment time and distance (respectively, *p*-value = 0.151, *p*-value = 0.330, and *p*-value = 0.590). Characteristics considered for meta-analysis of included studies are summarized in Table 1.

**Table 1:** Characteristics of studies included in the meta-analysis.

Author, year	Design	Gas	Distance (mm)	Time of exposure	Number of sockets		Log (CFU/ml) (S.D.)	
					Plasma	Control	Test	Control
Arguello-Sánchez et al. 2023 (54)	*Ex-vivo*	He	0	5 min.	10	10	2.88 (0.16)	7.17 (0.11)
Armand et al. 2019 (47)	*Ex-vivo*	He/O_2_	2 mm	8 min.	10	10	0.175 (0.35)	5.041 (0.65)
Asnaashari et al. 2022 (55)	*Ex-vivo*	He/O_2_	10 mm	10 min.	10	10	8.09 (0.18)	9.15 (0.26)
Kumar et al. 2023 (58)	*Ex-vivo*	He	2 mm	10 min.	21	21	0.95 (0.66)	4.76 (0.43)
Hufner et al. 2016 (49)	*Ex-vivo*	Ar/O_2_	3 mm	12 min.	10	20	4.18 (1.39)	6.11 (0.53)
Kerlikowski et al. 2020 (61)	*Ex-vivo*	Ar/O_2_	1–2 mm	12 min.	10	10	3.84 (1.38)	6.55 (0.56)
Li et al. 2015 (50)^a^	*Ex-vivo*	Ar/O_2_	10 mm	12 min.	10	10	0.00 (0.00)	7.30 (0.25)
Pan et al. 2013 (1)^a^	*Ex-vivo*	Ar/O_2_	5 mm	10 min.	10	10	0.00 (0.00)	7.00 (0.01)
Schaudinn et al. 2013 (60)	*Ex-vivo*	He/O_2_	/	30 min.	9	9	5.55 (2.70)	6.50 (2.90)
Kaya et al. 2014 (52)	*Ex-vivo*	He/O_2_	1 mm	5 min.	12	6	2.47 (2.29)	5.66 (0.61)

^a^ Studies were not included in the meta-analysis since zero means and SDs returned estimation error.

## 4 Discussion

The oral cavity consists of a wide number of microorganisms involved in endodontic and periodontal diseases, as well as dental caries (19). Following the pulp necrosis, oral bacteria colonize the root canals, initially in their planktonic phenotype, free of floating, and arranging in biofilm, attached to root canal walls (62). Biofilms are highly organized and complex communities of microorganisms, often multispecies, able to better resist to adverse conditions, such as nutrient deprivation, the host immune system and antimicrobials.

As expanding application of CAP as therapeutic mean in medicine, results from this systematic review and meta-analysis highlight a successful antimicrobial activity for both planktonic and biofilm phenotype (SMD = 4.51, 95% C.I. = 2.55–6.48, *p*-value < 0.001).

The complex anatomy of the root canal allows microorganisms to develop biofilm in internal sites, hardly reached by standard endodontic treatments. Therefore, the physical and chemical properties of CAP treatment may enhance the efficacy of traditional root canal disinfection methods (48, 50–52, 61).

The localization of biofilms on the walls of ramifications and isthmuses of the root canal poses difficulties in the removal of these persistent microorganisms, increasing the rate of endodontic treatment failure. Among biofilms, the exchange of resistance genes between different clinically relevant species is facilitated, causing the acceleration of the phenomenon of the antimicrobial resistance (63). Therefore, endodontic infections could be a reservoir for antimicrobial resistance.

The microbial communities associated with endodontic infections are heterogeneous, and their persistence in root canal infections is a major role in endodontic treatment failure. Among microbial oral communities, *E. faecalis* is one of the most frequent microorganisms isolated from patients with endodontic infections, especially in cases of reinfections (64). *E. faecalis* has a prolonged survival capacity and a morphology that facilitates its diffusion into dentinal tubules. Its virulence factors, such as surface adhesins, are responsible for the formation of a strong biofilm within the root canals (64). From the moment that the plasma jet could curve within the curved root canals, the plasma could reach any place inside the inner canal, inactivating hidden bacteria in places where current therapies cannot reach (33, 52). Furthermore, the reactive species produced by CAP are energized and move at a high speed, so they can more easily enter complex and thin root canals to kill bacteria. Compared to traditional disinfection methods, CAP treatment demonstrated variable results. Some studies reported higher efficacy compared to CHX (50, 56) or NaOCl (61), while others observed lower efficacy relative to NaOCl (49, 60), although a reduction in CFU was consistently noted compared to the control. Habib et al. (57) observed that CAP treatment was as effective as NaOCl irrigation. CAP demonstrated a similar reduction in bacterial metabolic activity and viability compared to NaOCl treatment, with the potential for fewer side effects in patients.

The present systematic review has summarized data from *in vitro* and *ex vivo* studies with similar objectives for a better understanding of the possible CAP applications in endodontics. A great variability in CAP treatment parameters and CAP effects has emerged, claiming for standardization of protocols for a possible future clinical application of plasma therapy in endodontics. The literature data demonstrated that the antimicrobial effect of CAP depended on time and distance. The most used distances were between 0 and 15 mm whereas the time of application was in the range of 1–15 min; only in a few works, plasma was applied for seconds. Moreover, CAP efficacy was greater against planktonic than biofilm associated bacteria (53). Wen et al. (53) found that in order to obtain a similar CFU reduction, 1 min of treatment was necessary for the planktonic phenotype and at least 5 min for the biofilm phenotype. These results were not confirmed from the meta-analysis results of *ex-vivo* experiments, where effect size did not correlate with both time and distance. These results should be read with caution as numerical information. Indeed, most of the studies lacked detailed information about the effective output power and energy, which might help replicate the treatment protocol. Indeed, short exposure time in close distance might be similar in results to larger treatment time, but to an increased distance. While for *in vitro* study, reproducibility is easier to achieve, for *ex vivo* model more variables are taken into account, such as complex anatomy, bacterial species and biofilm formation, which represent more source of confounding effect.

The uncertainty of these results must be addressed in future studies, in which a better understanding of these protocol variables should be investigated under proper study designs.

Most of the selected *ex vivo* studies tested the efficacy of plasma treatment either alone or in combination with other chemical compounds (48–53, 61). CAP treatment alone had a similar or greater effect than chemical treatments. Indeed, in Wen et al. (53) CHX groups exhibited a lower antibacterial activity than CAP. Surprisingly, CAP had a better effect compared to CAP-CHX combination, since CHX might scavenge the reactive species generated by CAP. Similar results were obtained when comparing the efficacy of CAP vs. conventional chemical agents (50, 51, 61), indicating the possibility of using CAP in alternative to chemical treatments.

Kerlikowski et al. (61) demonstrated that plasma/O_2_ treatment for 6 and 12 min achieved the highest CFU reduction rates, when compared to conventional chemical treatments with NaOCl, octenidine (OCT), CHX and the negative control with NaCl. Furthermore, 12 min of plasma/O_2_ application elicited the highest reduction of CFU; this data was also supported by SEM. Plasma/O_2_ combination with NaOCl, OCT, or CHX did not show a synergistic effect with respect to the only plasma/O_2_ treatments. In this study there was a further confirmation of the time-dependent antimicrobial activity of plasma.

Li et al. (50) demonstrated the superiority of CAP treatment over other endodontic medicaments, namely Ca(OH)_2_ paste, 2% CHX gluconate gel and a combination of both. Specifically, plasma treatment for 12 min resulted in biofilm destruction and bacterial killing as demonstrated by SEM images and CFU count, respectively. CSLM 3D images of *E. faecalis* biofilm showed that almost all bacteria were dead (50).

Wang et al. (51) investigated the time-dependent effectiveness of plasma treatment in comparison to other intracanal medicaments. Plasma microjet (PMJ) treatment was able to inactivate *E. faecalis* after 8 min and was effective even against *E. faecalis* reinfection, given that the application of PMJ for 40 min presented no reinfection. In detail, PMJ for 40 min had the same efficacy as the drug formocresol and better efficacy than camphor phenol and Ca(OH)_2_.

Ureyen Kaya et al. (52) compared the efficacy of CAP vs. Ozone and NaOCl in biofilm disinfection and concluded that CAP treatment has a greater cleaning efficacy than ozone in bacteria killing but lower than 2.5% of NaOCl.

Only in Ballout et al. (48) both plasma jet and DBD plasma irradiation did not show any effect against *E. faecalis* biofilm, given that the CFU count was similar to the one of negative controls in both coronal and apical part of the tooth. On the contrary, the positive control with NaOCl caused *E. faecalis* biofilm eradication as demonstrated by the total absence of CFU and by SEM images. The reason why both plasma jet and DBD plasma treatment modalities were not effective in biofilm disinfection was probably due to the low time of application (60 s) and by plasma source used. In fact, in most of the cited papers, plasma treatment was effective in CFU reduction only when applied for more than one minute. Furthermore, the type of gas used could influence the biofilm disinfection. A gas with He could be more effective than Ar as observed by Zhou et al. (59) who observed a significant reduction of biofilm starting from 1 min., although in this case an indirect approach is applied.

In an *in vitro* study Charoux et al. (44) used PAW as an antiseptic, showing a reduction in *E. faecalis* CFU following *in vitro* treatment. However, the characteristics of this technique make it less effective than gas, which has better capabilities in penetrating tortuous root canals as observed by Arguello-Sanchez et al. (54) in *ex vivo* study.

In summary, this systematic review demonstrates that CAP is a promising method in endodontic therapy whatever the approach used (mediated or not by liquid) and configuration of plasma sources applied (plasma jet-brush or plume, planar DBD). Different CAP devices have a better or similar effect in decreasing bacterial biofilms than conventional root canal disinfection methods based on rinsing the site of interest by a disinfecting solution. Furthermore, in most of the cited studies (1, 47, 50, 51, 53, 56, 57), CAP is effective in preventing *E. faecalis* reinfection, a condition which often leads to surgical therapy, probably due to plasma-generated reactive species which can successfully reach areas that are difficult to reach by traditional antiseptics. However, due to the heterogeneity among studies final conclusions on the CAP application modality as well as on plasma settings to obtain a proper root canal disinfection could not be drawn. Indeed, the considerable variety of plasma sources, settings, bacterial and biofilm models claim for the urgent need of standardization as building foundation for the clinical translation of CAP treatment. Although currently no types of plasma that can be used intraorally in patients, these studies provide suggestions on which setup parameters, biofilm models and examination methods should be used for further studies, so that at some point therapeutic recommendations or prerequisites for intraorally applicable plasma sources can be provided.

## 5 Limitations and future perspectives

A limitation of this study is the variety of devices used to produce CAP; furthermore, in this work only *in vitro* and *ex-vivo* experiments were analyzed, but none of *in vivo* studies were present. Moreover, there are not enough studies to compare the efficacy of the different methods of plasma administration, because only a few have analyzed the indirect method, such as the delivery of plasma activated means. This currently makes it impossible currently to establish an appropriate clinical protocol. Despite the limitations, CAP has shown efficacy against endodontic microorganisms in both *in vitro* and in *ex-vivo* studies, with an action similar to that observed in conventional methods, without damaging hard tissues of teeth as observed by Wen et al. (53). Regarding the effect of CAP on the oral mucosa, Jablonowski et al. (65) observed how treatment with CAP is well tolerated in the short term experiments by the murine model, while Evert et al. (66) observed that repeated exposure to CAP of the oral mucosa over 12 months is not carcinogenic in mice, confirming the safety of the treatment also for long term exposure. The results reported by Jablonowski et al. (65) and Evert et al. (66) demonstrated the safety of these CAP devices, making them a promising technique for future use in patients undergoing root canal treatment. Consequently, the focus on the use of CAP and the encouraging results observed in numerous studies could drive the progress of technical research, allowing the creation of increasingly advanced devices to enable the future application of CAP for the treatment of root canals.
