# Supplementary material for: Disinfection of dental root canals by cold atmospheric plasma: a systematic review and meta-analysis of dental biofilm
Source: Front Oral Health. 2024 Dec 3;5:1483078. doi: 10.3389/froh.2024.1483078 (PMC11650461; doi:10.3389/froh.2024.1483078)
Supplement: Supplementary file 1 [file Table1.docx]

Supplementary Material

# Supplementary Tables

**Supplementary Table 1:** Search Strategy

| **Databases** | **Search String** |
| --- | --- |
| **PubMed:** | (“COLD ATMOSPHERIC PLASMA” OR “COLD PLASMA” OR “LOW TEMPERATURE PLASMA” OR “KINPEN MED” OR “NON-THERMAL ATMOSPHERIC PRESSURE PLASMA” OR “NONTHERMAL ATMOSPHERIC PRESSURE PLASMA” OR “COLD PHYSICAL PLASMA” OR “PLASMA MEDICINE” OR PAM OR CAP OR “PLASMA ACTIVATED MEDIUM” OR “COLD ATMOSPHERIC-PRESSURE PLASMA” OR “PLASMA GASES” OR “PLASMA ACTIVATED LIQUID” OR “COLD ARGON PLASMA” OR “PLASMA JET” OR “AIR PLASMA”) AND (BIOFILM OR BIOFILMS OR “ORAL BIOFILM” OR “ORAL MICROORGANISM*” OR “PERIODONTAL PATHOGEN*” OR “BIOFILM COLONIZATION*” OR “DENTAL PLAQUE” OR “DENTAL DEPOSIT*” OR “MATERIA ALBA” OR “PERIODONTAL DISEASE*” OR PERIODONT* OR PARODONT* OR “PYORRHEA ALVEOLARIS” OR “PERI-IMPLANT DISEASE*” OR PERI-IMPLANT* OR PERIIMPLANT* OR PERI IMPLANT*) |
| **Web of Science:** | Set for search in all Databases by Topic under Documents (“cold atmospheric plasma” OR “cold plasma” OR “low temperature plasma” OR “kinpen med” OR “non-thermal atmospheric pressure plasma” OR “non thermal atmospheric pressure plasma” OR “cold physical plasma” OR “plasma medicine” OR pam OR cap OR “plasma activated medium” OR “cold atmospheric-pressure plasma” OR “plasma gases” OR “plasma activated liquid” OR “cold argon plasma” OR “plasma jet” OR “air plasma”) AND (biofilm OR biofilms “oral biofilm” OR “oral microorganism*” OR “periodontal pathogen*” OR “biofilm colonization*” OR “dental plaque” OR “dental deposit*” OR “materia alba” OR “periodontal disease*” OR periodont* OR parodont* OR “pyorrhea alveolaris” OR “peri-implant disease*” OR peri-implant* OR periimplant* OR periimplant*) |
| **Scopus:** | ("cold atmospheric plasma" OR  "cold plasma"  OR  "low temperature plasma"  OR  "kinpen med"  OR  "non-thermal atmospheric pressure plasma"  OR  "non thermal atmospheric pressure plasma"  OR  "cold physical plasma"  OR  "plasma medicine"  OR  pam  OR  cap  OR  "plasma activated medium"  OR  "cold atmospheric-pressure plasma"  OR “plasma gases” OR  "plasma activated liquid"  OR  "cold argon plasma"  OR  "plasma jet"  OR  "air plasma" ) AND  (biofilm  OR biofilms OR "oral biofilm"  OR  "oral microorganism*"  OR  "periodontal pathogen*"  OR  "biofilm colonization*"  OR  "dental plaque"  OR  "dental deposit*"  OR  "materia alba"  OR  "periodontal disease*"  OR  periodont*  OR  parodont*  OR  "pyorrhea alveolaris"  OR  "peri-implant disease*"  OR  peri-implant*  OR  periimplant*  OR  peri  AND implant* ) |

**Supplementary table 2.** Bias risk assessment and methodological quality for *in vitro* studies according to Perrotti et al., 2021. IT1- condition of bacterial culture before experimentation; IT2- condition of bacterial culture during treatment; IT3- description of methodology to evaluate outcomes; IT4- case-control description; IT5- multiple experiments performed; IT6- descriptions of plasma settings and devices. ✓- Yes; – No; U – Unclear; N/A – Not applicable

|  | IT1 | IT2 | IT3 | IT4 | IT5 | IT6 | %yes/risk | Risk of Bias |
| --- | --- | --- | --- | --- | --- | --- | --- | --- |
| Avukat E et al. 2023 (39) | ✓ | ✓ | ✓ | ✓ | ✓ | ✓ | 100.00 | Low |
| Cao Y et al. 2011 (33) | ✓ | ✓ | ✓ | ✓ | ✓ | ✓ | 100.00 | Low |
| Chang Y et al., 2016 (32) | ✓ | ✓ | ✓ | - | - | ✓ | 66.66 | Low |
| Charoux C et al. 2020 (44) | ✓ | ✓ | ✓ | ✓ | ✓ | ✓ | 100.00 | Low |
| Delben JA et al. 2014 (42) | ✓ | ✓ | ✓ | ✓ | ✓ | ✓ | 100.00 | Low |
| Hirano Y et al. 2019 (36) | ✓ | ✓ | ✓ | ✓ | ✓ | ✓ | 100.00 | Low |
| Jablonowski L et al. 2013 (37) | ✓ | ✓ | ✓ | ✓ | ✓ | ✓ | 100.00 | Low |
| Leite P et al. 2021 (41) | ✓ | ✓ | ✓ | - | ✓ | ✓ | 83.33 | Low |
| Liu T et al. 2017 (45) | ✓ | ✓ | ✓ | - | ✓ | ✓ | 83.33 | Low |
| Maisch T et al. 2012 (40) | ✓ | ✓ | ✓ | ✓ | ✓ | ✓ | 100.00 | Low |
| Matthes R et al. 2015 (43) | ✓ | ✓ | ✓ | ✓ | ✓ | ✓ | 100.00 | Low |
| Theinkom F et al. 2019 (35) | ✓ | ✓ | ✓ | ✓ | ✓ | ✓ | 100.00 | Low |
| Yoo E et al. 2020 (46) | ✓ | ✓ | ✓ | ✓ | ✓ | ✓ | 100.00 | Low |
| Zhou X et al. 2010 (34) | ✓ | - | ✓ | - | - | ✓ | 50.00 | Moderate |
| Zhu M et al. 2023 (38) | ✓ | ✓ | ✓ | ✓ | ✓ | ✓ | 100.00 | Low |

**Supplementary Table 3:** Bias risk assessment and methodological quality for ex-vivo studies according to Sarkis-Onofre et al., 2014. Q1- Was there a randomization of extracted teeth when they were divided between groups? Q2- The extracted teeth were free of caries or restorations? Q3- The researched materials were used according to the manufacturers’ instructions? Q4- Did the sample used have similar dimensions? Q5- The endodontic treatment of the samples was performed by a single operator? Q6- Were defined the characteristic of bacteria cultivation? Q7- Was the operator of the mechanical test blind to the type of sample tested? Q8- Was plasma protocol descripted? ✓- Yes; – No; U – Unclear; N/A – Not applicable.

|  | Q1 | Q2 | Q3 | Q4 | Q5 | Q6 | Q7 | Q8 | % yes/risk | Risk of Bias |
| --- | --- | --- | --- | --- | --- | --- | --- | --- | --- | --- |
| Arguello-Sánchez R et al. 2023 (54) | ✓ | ✓ | ✓ | ✓ | N/A | ✓ | - | ✓ | 75.00 | Low |
| Armand A et al. 2019 (47) | ✓ | - | ✓ | - | - | ✓ | - | ✓ | 50,00 | Moderate |
| Asnaashari M et al. 2022 (55) | ✓ | ✓ | ✓ | ✓ | N/A | ✓ | - | ✓ | 75.00 | Low |
| Ballout H et al. 2018 (48) | ✓ | ✓ | ✓ | ✓ | - | ✓ | - | ✓ | 75,00 | Low |
| Du T et al. 2012 (56) | ✓ | ✓ | ✓ | ✓ | N/A | ✓ | - | ✓ | 75,00 | Low |
| Habib M et al. 2014 (57) | ✓ | ✓ | ✓ | ✓ | N/A | ✓ | - | ✓ | 75,00 | Low |
| Huefner A et al. 2017 (49) | ✓ | ✓ | ✓ | - | - | - | - | ✓ | 50,00 | Moderate |
| Kaya B et al. 2014 (52) | ✓ | ✓ | ✓ | ✓ | - | ✓ | - | ✓ | 75,00 | Low |
| Kerlikowski A et al. 2020 (61) | ✓ | ✓ | ✓ | - | - | ✓ | - | ✓ | 62,50 | Low |
| Kumar P et al. 2023 (58) | ✓ | ✓ | ✓ | ✓ | N/A | ✓ | - | ✓ | 75,00 | Low |
| Li Y et al. 2015 (50) | ✓ | - | ✓ | - | - | ✓ | - | ✓ | 50,00 | Moderate |
| Pan J et al. 2013 (1) | ✓ | - | ✓ | - | - | ✓ | - | ✓ | 50,00 | Moderate |
| Schaudinn C et al. 2013 (60) | ✓ | - | ✓ | - | - | ✓ | - | ✓ | 50,00 | Moderate |
| Wang R et al. 2011 (51) | ✓ | ✓ | ✓ | - | - | ✓ | - | ✓ | 62,50 | Low |
| Wen Y et al.  2022 (53) | ✓ | ✓ | ✓ | ✓ | - | ✓ | - | ✓ | 75,00 | Low |
| Zhou X et al. 2016 (59) | ✓ | ✓ | ✓ | ✓ | N/A | ✓ | - | ✓ | 75,00 | Low |

**Supplementary table 4:** List of excluded studies and reason for exclusion

| **Study** | **Reasons for Exclusion** |
| --- | --- |
| Yesildal 2022 | No antimicrobial capacity of CAP was tested in this study |
| Yingguang 2011 | the bacterium is not contextualized in the root canals |

**Supplementary Table 5:** *in vitro* studies. ↓ diminution respect control; ↓↓ significative diminution respect control; AA: airborne acoustic ultrasound; Ampho B: amphotericin B; Ar: Argon; CHX: chlorhexidine; CLSM: Confocal Laser Scanning Microscopy; cPAW: PAW generated from cold plasma; CPR: Ciprofloxacin; CW: non-treated water; DPT: daptomycin; He: helium; NaOCl: Sodium hypochlorite; Nys: Nystatin; PAW: plasma activated water; SEM: Scanning Electron Microscopy; tPAW: PAW generated from thermal plasma; Untr: Untreated

| **Authors** | **Microorganism** | **Study design** | **Biofilm**  **(coltivation duration)** | **Plasma treatment** | **Primary Outcomes** | **Results** |
| --- | --- | --- | --- | --- | --- | --- |
| Avukat E et al. 2023 (39) | *C. albicans* | **G1**= Control (Untr)  **G2**= 80% He-20% O_2_ plasma treatment  **G3**= 85% He--15% O_2_ plasma treatment  **G4**= 90% He--10% O_2_ plasma treatment  **G5**= 100% He-plasma treatment | 48h | Direct (low pressure RF sputtering)  **Biofilm substrate:**  Dry biofilm on PMMA discs | **MTT**  **SEM** | **MTT**  **G2**= ↓↓  **G3**= ↓↓  **G4**= ↓↓  **G5**= ↓↓  **SEM**  **G2**= ↓↓  **G3**= ↓↓  **G4**= ↓↓  **G5**= ↓↓ |
| Cao Y et al. 2011 (33) | *E. faecalis* | **G1**= Control (Untr)  **G2**= 5 min 98% He-2%O_2_ plasma | 48h | Direct (plasma Jet)  **Biofilm substrate:**  Agar and dry biofilm on porous sterile cellulose membrane | **CFU**  **SEM** | **CFU**  **G2**=↓↓  **SEM**  **G2**=↓↓ |
| Chang Y et al. 2016 (32) | *E. faecalis* | **G1**=Control (Untr)  **G2**= 1 min ambient air plasma  **G3**= 2 min ambient air plasma  **G4**= 5 min ambient air plasma  **G5**= 10 min ambient air plasma  **G6**= 15 min ambient air plasma | / | Direct (planar DBD)  **Biofilm substrate:**  Dry biofilm on cover glass | **CFU**  **SEM** | **CFU**  **G2**= ↓  **G3**= ↓↓  **G4**= ↓↓  **G5**= ↓↓  **G6**= ↓↓  **SEM**  **G3**= ↓↓ |
| Charoux C et al. 2020 (44) | *E. coli* | **G1**= Control (treated with distilled water)  **G2**= CW treatment  **G3**= AA treatment  **G4**= cPAW treatment  **G5**= tPAW (feed: ambient air) treatment  **G6**= AA+cPAW  **G7**= AA+tPAW | 72h | Indirect (plasma jet or thermal plasma beam)  **Biofilm substrate:**  Solution in sterile distilled water | **CFU** | **CFU**  **G2**= ↓↓  **G3**= ↓↓  **G4**= ↓↓  **G5**= ↓↓  **G6**= ↓↓  **G7**= ↓↓ |
| Delben JA et al. 2014 (42) | *C. albicans* | **G1**= Control (Untr)  **G2**= Ar gas application (3mm)  **G3**= Ar gas application (10mm)  **G4**= Ar plasma application (3mm)  **G5**= Ar plasma application (10mm) | 72h | Direct (plasma jet)  **Biofilm substrate:**  Agar | **Growth Inhibition Zone**  **CLSM** | **Growth Inhibition Zone**  **G2**= **-**  **G3**= **-**  **G4**= **↑**  **G5**= **↑**  **CLSM**  **G2**= **↓**  **G3**= ↓  **G4**= ↓  **G5**= ↓ |
| Hirano Y et al. 2019 (36) | *S. mutans*  *E. faecalis* | **G1**=0, 3, 5, 7 min. ambient air plasma treatment (*S mutans*)  **G2**=0, 3, 5, 7 min. ambient air plasma treatment (*E. faecalis*) | 24 h (S. mutans) 18 h (E. faecalis) | Direct (plasma jet)  **Biofilm substrate:** Suspension of bacteria in PBS | **CFU** | **G1**= 3 ↓, 5 ↓↓, 7 ↓↓ min.  **G2**=0 ↓, 3 ↓↓, 5 ↓↓, 7 ↓↓ min. |
| Jablonowski L et al. 2013 (37) | *E. faecalis* | **G1**= Control (NaCl)  **G2**=CHX  **G3**=NaOCl  **G4**=Ar Plasma | 2h | Direct (Plasma Jet)  **Biofilm substrate:**  Plastic blocks | **CFU** | **G2**=↓↓  **G3**=↓↓  **G4**=↓↓ |
| Leite P et al. 2021 (41) | *C. albicans* SC5314, P29 | **C. albicans SC5314 (biofilm 24h, 48h)**  **G1**= Control  **G2**= Nys  **G3**= Ampho B  **G4**= 99.5% He Plasma  **C albicans P29 (biofilm 24h, 48h)**  **G1**= Control  **G2**= Nys  **G3**= Ampho B  **G4**= 99.5% He Plasma | 24h, 48h | Direct (plasma jet)  **Biofilm substrate:**  Biofilm on 96 well plate | **CFU** | **CFU**  **C.albicans SC5314 24h**  **G2**= ↓  **G3**= ↓↓  **G4**= ↓↓  **C.albicans SC5314 48h**  **G2**= ↓  **G3**= ↓↓  **G4**= ↓↓  **C.albicans P29 24h**  **G2**= ↓↓  **G3**= ↓↓  **G4**= ↓↓  **C.albicans P29 48h**  **G2**= ↓↓  **G3**= ↓↓  **G4**= ↓↓ |
| Liu T et al. 2017 (45) | *S. mutans,*  *S. sanguinis* | **G1**= Ar-1% O2 Plasma effect on S.mutans  **G2**= Ar-1% O_2_ Plasma effect on S. sanguinis  **G3**= Ar-1%O_2_ Plasma effect on S.mutans+ S. sanguinis | 7 days | Direct (plasma brush)  **Biofilm substrate:**  Biofilm on 48 well plate covered with PBS | **MTT** | **MTT**  **G1**= ↓↓  **G2**= ↓↓  **G3**= ↓↓ |
| Maisch T et al. 2012 (40) | *C. albicans* | **G1**= Control (Untr)  **G2**= 20 sec. ambient air plasma  **G3**= 40 sec. ambient air plasma  **G4**= 60 sec. ambient air plasma  **G5**= 120 sec. ambient air plasma  **G6**= 5 min. ambient air plasma  **G7**= 6 min. ambient air plasma  **G8**= 7 min. ambient air plasma  **G9**= 8 min. ambient air plasma  **G10**= 9 min. ambient air plasma  **G11**= 10 min. ambient air plasma | 24h | Direct (surface micro discharge)  **Biofilm substrate:**  Dry biofilm on agar | **CFU** | **CFU**  **G1**= ↓  **G2**= ↓  **G3**= ↓  **G4**= ↓  **G5**= ↓  **G6**= ↓  **G7**= ↓  **G8**= ↓↓  **G9**= ↓↓  **G10**= ↓↓  **G11**= ↓↓ |
| Matthes R et al. 2015 (43) | *C. albicans* | **G1**= Control (NaCL) 2, 7, 16 days biofilm  **G2**= Ar gas 2, 7, 16 days  **G3**= Ar Plasma 2, 7, 16 days  **G4**= Ar-1%O _2_ plama 2 days  **G5**=CHX 2, 7, 16 days  **G6**= NaOCl 7, 16 days biofilm | 2,7,16 days | Direct (volume DBD)  **Biofilm substrate:**  Dry biofilm deposited on PMMA discs | **CFU** | **G2**= 2 ↓, 7 ↓↓, 16 ↓ days biofilm  **G3**= 2↓↓, 7 ↓, 16 ↓ days biofilm  **G4**=2↓↓  **G5**= 2↓↓, 7 ↓, 16 ↓ days biofilm  **G6**= 7 ↓↓, 16 ↓↓, days biofilm |
| Theinkom F et al. 2019 (35) | *E. faecalis* | **G1**= Control (Untr)  **G2**= 1 min. ambient air plasma  **G3**= 3 min. ambient air plasma  **G4**= 5 min. ambient air plasma  **G5**= 10 min. ambient air plasma | 24h | Direct (surface micro-discharge)  **Biofilm substrate:**  Agar plates or dry biofilm growth on PS petri dishes | **CFU** | **CFU**  **G2**= ↓  **G3**= ↓  **G4**=↓↓  **G5**= ↓↓ |
| Yoo E et al. 2020 (46) | *S. mutans* | **G1**= Control (Untr)  **G2**= 30 sec. N_2_ plasma  **G3**= 60 sec. N_2_ plasma  **G4**= 120 sec. N_2_ plasma | 24h | Direct (plasma jet)  **Biofilm substrate:**  Dry biofilm on hydroxyapatite | **CFU**  **SEM**  **CLSM** | **CFU**  **G2**= ↓  **G3**= ↓↓  **G4**= ↓↓  **SEM**  **G2**= ↓  **G3**= ↓↓  **G4**= ↓↓  **CLSM**  **G2**= ↓  **G3**= ↓↓  **G4**= ↓↓ |
| Zhou X et al. 2010 (34) | *E. faecalis* | **G1**= Control (Untr)  **G2**= 2 min. He-1%O_2_ plasma  **G3**= 4 min. He-1%O_2_ plasma  **G4**= 6 min. He-1%O_2_ plasma  **G5**= 8 min. He-1%O_2_ plasma  **G6**= 10 min He-1%O_2_ plasma  **G7**= 12 min He-1%O_2_ plasma | 72h | Direct (plasma jet)  **Biofilm substrate:**  Dry biofilm inside a simulated root canal | **CFU** | **CFU**  **G2**=↓↓  **G3**= ↓↓  **G4**= ↓↓  **G5**= ↓↓  **G6**= ↓↓  **G7**= ↓↓ |
| Zhu M et al. 2023 (38) | *E. faecalis* | **G1**= Control (PBS)  **G2**=2.5% NaOCl  **G3**=2% CHX  **G4**=PMBs  **G5**=PMBS+US | 7 days | Direct ( PMBs)  **Biofilm substrate:**  Biofilm on dentin block | **SEM**  **CLSM** | **SEM**  **G2**=↓↓  **G3**=↓  **G4**=↓↓  **G5**=↓↓  **CLSM**  **G2**=↓↓  **G3**=↓  **G4**=↓↓  **G5**=↓↓ |

**Supplementary Table 6:** *ex vivo* studies. ↑ upregulation respect control ↑↑ significative upregulation respect control ↓ diminution respect control; ↓↓ significative diminution respect control; ↓↓↓ significative diminution respect positive; Ar: Argon; CAP: Cold atmospheric pressure plasma; CFU: Colony Forming Units; CHX: Chlorhexidine; CLSM: Confocal Laser Scanning Microscopy; He: Helium; LDCP: Liquid Direct Cold Plasma; LTAPP: Low-temperature atmospheric pressure plasma; NaCl: sodium chloride; NaOCl: Sodium hypochlorite; NTP: Non – thermal plasma; O2: Oxygen; OCT: Octenidine; PDT: photosesitizer and laser treatment; PAW: Plasma activated water; SEM: Scanning Electron Microscopy; TEM: Transmission Electron Microscopy; PMB: plasma-loaded microbubbles; US: ultrasound.

| **Authors** | **Microorganism** | **Study design** | **Biofilm (coltivation duration)** | **Plasma treatment** | **Primary Outcomes** | **Results** |
| --- | --- | --- | --- | --- | --- | --- |
| Arguello-Sánchez R et al. 2023 (54) | *E. faecalis* | **G1**= Control (Untr) (n=10)  **G2**= 5 min. He Plasma (5 mm) (n=10)  **G3**= 5 min. PAW (sterile distilled water)  **G4**= 5 min. He Plasma (0 mm) (n=10)  **G5**= 5 min. NaOCl (0.6%) (n.10)  **G6**= 1 min. NaOCl (5.25%) (n=10)  **G7**=5 min. NaOCl (5.25%) (n=10) | 3 weeks | Direct (plasma jet); Indirect | **CFU** | **CFU**  **G2**= ↓  **G3**= ↓  **G4**= ↓↓  **G5**= ↓  **G6**= ↓↓  **G7**= ↓↓ |
| Armand A et al. 2019 (47) | *E. faecalis* | **G1**= control (n=10)  **G2**=He plasma 4, 6, 8 min. (n=10)  **G3**= He-0.5% O_2_ plasma 4, 6, 8 min.(n=10)  **G4**= PDT (n=5) | 4 weeks | Direct (plasma jet) | **CFU**  **SEM** | **CFU**  **G2**= 4↓, 6↓↓, 8↓↓  **G3**= 4↓↓, 6↓↓, 8↓↓  **G4**= ↓↓  **SEM**  **G2**= ↓↓  **G3**= ↓↓  **G4**= ↓↓ |
| Asnaashari M et al. 2022 (55) | *E. faecalis* | **G1**= Control of calcium hydroxide (n=10)  **G2**= Calcium hydroxide (n.10)  **G3**= Control of TAP (n=10)  **G4**= TAP (n.10)  **G5**= Control of Plasma (n=10)  **G6**=95% He-5%O_2_ plasma (n=10) | 3 weeks | Direct (plasma jet) | **CFU** | **CFU**  **G2**=**↑**  **G4**=**↓↓**  **G6**=**↓↓** |
| Ballout H et al. 2018 (48) | *E. faecalis* | **G1**= Control (0.9% NaCl) (n=10)  **G2**= Ar Plasma Jet (CAP) (n=10)  **G3**= Ambient Air DBD (CAP) (n=10)  **G4**= PDT (n=10)  **G5**= NaOCl (n=10) | 6 days | Direct (plasma jet and DBD) | **CFU**  **SEM** | **CFU**  **G2**= ↓  **G3**= ↓  **G4**= ↓  **G5**= ↓↓  **SEM**  **G2**= ↓  **G3**= ↓  **G4**= ↓  **G5**= ↓↓ |
| Du T et al 2012 (56) | *E. faecalis* | **G1**= 5 min. NaCl (n=8)  **G2=** 10 min. NaCl (n=8)  **G3**= 15 min. NaCl (n=8)  **G4**= 5 min. 2% CHX (n=8)  **G5**= 10 min. 2% CHX (n=8)  **G6**= 15 min. 2% CHX (n=8)  **G7**= 5 min. He-O_2_ Plasma jet (n=8)  **G8**= 10 min. He-O_2_ Plasma jet (n=8)  **G9**= 15 min. He-O_2_ Plasma jet (n=8) | 2 days | Direct (plasma jet) | **CFU** | **CFU**  **G4**= **↓↓**  **G5**= **↓↓**  **G6**= **↓↓**  **G7**= **↓↓**  **G8**= **↓↓**  **G9**= **↓↓** |
| Habib M et al. 2014 (57) | *E. faecalis* | **G1**= Control (PBS) (8)  **G2**= 6% NaOCl (8)  **G3**= Ar-10% O_2_ Plasma (8) | 2 days | Direct (brush-shaped plasma jet) | **CFU**  **MTT**  **XTT** | **CFU**  **G2**= **↓↓**  **G3**= ↓↓  **MTT**  **G2**= **↓↓**  **G3**= **↓↓**  **XTT**  **G2**= **↓↓**  **G3**= **↓↓** |
| Huefner A et al. 2017 (49) | *E. faecalis* | **G1**= Control 6 min. (saline) (n=20)  **G2**= Ar plasma 6 min. (n=10)  **G3**=Ar-1% O_2_ 6 min.(n=10)  **G4**= Ar Plasma 6 min. (n=10)  **G5**= Ar-1% O_2_ 6 min. (n=10)  **G6**=NaOCl 6 min. (n=10)  **G7**=NaOCl 12 min. (n=10)  **G8**=NaOCl+ Ar plasma 12 min. (n=10)  **G9**=NaOCl+ Ar-1%O_2_ plasma 12 min. (n=10)  **G10**=Ar Plasma 12 min. (n=10)  **G11**= Ar-1% O_2_ 12 min. (n=10) | 1 week | Direct (plasma jet) | **CFU** **SEM** | **CFU**  **G2**= ↓↓  **G3**= ↓↓  **G4**= ↓↓  **G5**= ↓↓  **G6**= ↓↓  **G7**= ↓↓  **G8**= ↓↓  **G9**= ↓↓  **G10**= ↓↓  **G11**= ↓↓  **G7**=↓ vs G6  **G10**= ↓ vs G4  **G11**= ↓↓ vs G5  **SEM**  **G2**= ↓↓  **G3**=↓↓  **G4**= ↓↓  **G5**=↓↓  **G6**= ↓↓  **G7**= ↓↓  **G8**= ↓↓  **G9**= ↓↓  **G10**= ↓↓  **G11**= ↓↓ |
| Kaya B et al. 2014 (52) | *E. faecalis* | **G1**= Control (saline) (n=6)  **G2**= NaOCl (n=12)  **G3**= Ozone (n=12)  **G4**= LTAPP (n=12) | 4 weeks | Direct (plasma jet) | **CFU** | **CFU**  **G2** = ↓↓  **G3** = ↓  **G4** = ↓↓ |
| Kerlikowski A et al. 2020 (61) | *C. albicans* | **G1**= Control 6 min. (0.9% NaCl) (n=10)  **G2**= Gas 6 min. (n=10)  **G3**= 5.25% NaOCl 6 min. (n=10)  **G4**= 2% CHX 6 min. (n=10)  **G5**= 0.1% OCT 6 min. (n=10)  **G6**= Ar-1% O_2_ plasma6 min. (n=10)  **G7**= Control 20 min. (NaCl) (n=10)  **G8**= Gas 20 min. treatment (n=10)  **G9**=5.25% NaOCl 20 min. (n=10)  **G10**= 2% CHX 20 min. (n=10)  **G11**= 0.1% OCT 20 min. (n=10)  **G12**= Ar-1% O_2_ plasma20 min. (n=10)  **G13**= 5.25% NaOCl + Ar-1% O_2_ plasma20 min. (n=10)  **G14**= 2% CHX + Ar-1% O_2_ plasma 20 min. (n=10)  **G15**= OCT+ Ar-1% O_2_ plasma 20 min. (n=10) | 1 week | Direct (plasma jet) | **CFU SEM** | **CFU**  **G2**= ↓ vs G1  **G3**= ↓↓ vs G1  **G4**= ↓↓ vs G1  **G5**= ↓↓ vs G1  **G6**= ↓↓ vs G1  **G8**= ↓ vs G1  **G9**= ↓↓ vs G1 **G10**= ↓↓ vs G7  **G11**= ↓↓ vs G7  **G12**= ↓↓ vs G7  **G13**= ↓↓ vs G7  **G14**= ↓↓ vs G7  **G15**= ↓↓ vs G7  **SEM**  **G6**= ↓↓ vs G1  **G13**=↓↓ vs G1  **G14**= ↓↓ vs G1  **G15**= ↓↓ vs G1 |
| Kumar P et al. 2023 (58) | *E. faecalis* | **G1**=Control (0.9% NaCl) (n.21)  **G2**= 2 min. treatment, He plasma (n.21), QMix (n.21), 3% NaOCl (n.21)  **G3**=5 min. treatment, He plasma (n.21), QMix (n.21), 3% NaOCl (n.21) | 1 week | Direct (plasma jet) | **CFU** | **G2**= CAP ↓, QMix ↓↓, NaOCl ↓↓  **G3**= CAP↓↓, QMix ↓↓, NaOCl↓↓ |
| Li Y et al. 2015 (50) | *E. faecalis* | **G1**= Control (Untr) (n=10)  **G2**= Ar-2%O_2_ Plasma 3 min. (n=10)  **G3**= Ar-2%O_2_ Plasma 6 min. (n=10)  **G4**= Ar-2%O_2_ Plasma 9 min. (n=10)  **G5**= Ar-2%O_2_ Plasma 12 min. (n=10)  **G6**= Ca(OH)_2_ 7 days (10)  **G7**= 2% CHX 7 days (n=10)  **G8**= Ca(OH)_2_/CHX 7 days (n=10) | 3 weeks | Direct (plasma jet) | **CFU** **SEM**  **CLSM** | **CFU G2**=↓  **G3**=↓  **G4**=↓  **G5**=↓↓  **G6**=↓  **G7**=↓  **G8**=↓  **SEM**  **G5**=↓↓  **CLSM**  **G5**=↓↓ |
| Pan J et al. 2013 (1) | *E. faecalis* | **G1**= Control (Untr) (n=10)  **G2**= 2 min. Ar-2%O_2_ plasma (n=10)  **G3**= 4 min. Ar-2%O_2_ plasma (n=10)  **G4**= 6 min. Ar-2%O_2_ plasma (n=10)  **G5**= 8 min. Ar-2%O_2_ plasma (n=10)  **G6**= 10 min. Ar-2%O_2_ plasma (n=10)  **G7**= positive control (Ca(OH)_2_) (n=10) | 1 week | Direct (plasma jet) | **CFU** **SEM**  **CLSM** | **CFU**  **G2**= ↓  **G3**=↓  **G4**= ↓  **G5**= ↓↓↓  **G6**= ↓↓↓  **G7**= ↓  **SEM**  **G6**= ↓↓  **CLSM**  **G6**= ↓↓ |
| Schaudinn C et al. 2013 (60) | Biofilm, unspecified | **G1**= Control (0.9% NaCl) (n=9)  **G2**= He-1% O_2_ Plasma (n=9)  **G3**= 6% NaOCl (n=9) | / | Direct (plasma jet) | **CFU**  **CLSM** | **CFU**  **G2**=↓↓  **G3**= ↓↓  **CLSM**  **G2**=↓↓  **G3**= ↓↓ |
| Wang R et al. 2011 (51) | *E. faecalis* | **G1**= 2 min. Ar-2% O_2_ plasma (n=6)  **G2**= 4 min. Ar-2% O_2_ (n=6)  **G3**= 6 min. Ar-2% O_2_ plasma (n=6)  **G4**= 8 min. Ar-2% O_2_ plasma (n=6)  **G5**= Control (gas) (n=5)  **G6**= Ar-2% O_2_ plasma (8,10,20, 30, 40 min.) (n=5)  **G7**=CP (n=5)  **G8**= Ca(OH)_2_ (n=5)  **G9**= FC (n=5) | 1 week | Direct (plasma jet) | **Inactivation rate**  **Re-infection rate SEM** | **Inactivation rate**  **G1**= ↑↑  **G2**= ↑↑  **G3**= ↑↑  **G4**= ↑↑  **Re-infection rate**  **G8**=8 min.↓, 10 min. ↓, 20 min. ↓↓, 40 min. ↓↓  **G9**= ↓↓  **G10**= ↓↓  **G11**= ↓↓  **SEM**  **G8**= 8 min.↓, 30 min. ↓↓ |
| Wen Y et al. 2022 (53) | *E. faecalis* | **G1**= Control (0.9% NaCl) (n=5)  **G2**= LDCP (n=5)  **G3**= 0.9% NaCl + 0.2% CHX (n=5)  **G4**= LDCP+0.2% CHX (n=5) | 4 weeks | Direct-liquid discharge cold plasma (root canal filled with 0.9% NaCl) | **CFU**  **CLSM**  **SEM** | **CFU**  **G2**=↓↓  **G3**=↓↓  **G4**=↓↓  **CLSM**  **G2**=↓↓  **SEM**  **G2**=↓↓ |
| Zhou X et al. 2016 (59) | *E. faecalis* | **G1**= control (n=6)  **G2**= 1 min. He+H_2_O_2_ plasma treatment without bubbling (n=6)  **G3**= 2 min He+H_2_O_2_ plasma treatment without bubbling (n=6)  **G4**= 4 min. He+H_2_O_2_ plasma treatment without bubbling (n=6)  **G5**= 1 min He+H_2_O_2_ plasma treatment with bubbling (n=6)  **G6**= 2 min He+H_2_O_2_ plasma treatment with bubbling (n=6)  **G7**= 4 min. He+H_2_O_2_ plasma treatment with bubbling (n=6) | 1 week | Indirect (plasma jet fed with He and vapours of H_2_O_2_) | **CFU** | **CFU**  **G2**= ↓↓  **G3**= ↓↓  **G4**= ↓↓  **G5**= ↓↓  **G6**= ↓↓  **G7**= ↓↓ |

**Supplementary Table 7:** CAP treatment parameters. Ar: Argon; CAP: Cold Atmospheric pressure Plasma; DBD: Dielectric Barrier Bischarge; SMDP: Surface Micro Discharge Plasma; He: Helium; L/min: Liters per minutes; N/P: Not provided; O2: oxygen; scm: Standard Cubic Centimeters per minute; slm: Standard liter per minute.

| **Authors** | **Plasma Device Description** | **Pulse frequency** | **Pulse** | **Gas** | **Flow rate** | **Plasma temperature** | **Application distance** | **Application time** | **Power** | **Manufacturer** |
| --- | --- | --- | --- | --- | --- | --- | --- | --- | --- | --- |
| Arguello-Sánchez R et al. 2023 (54) | Plasma jet | 1.356 kHz | N/P | He | 0.5 LPM | <40 °C | 0-5mm | 5 min. | 20W | N/P |
| Armand A et al. 2019 (47) | Plasma jet | 25 kHz | 5 kV | He  and He+0.5% O_2_ | 4 slm  and 20 scm | < 40 °C after 4,  6 and 8 min plasma exposure | 2 mm | 4 min treatment 6 min treatment 8 min treatment | N/P | Home-made device |
| Asnaashari M et al. 2022 (55) | Plasma jet | 14 kHz | 8 kV0.5 % O_2_ | He + 0.5 % O_2_ | N/P | N/P | 10mm | 10 min. | N/P | N/P |
| Avukat E et al. 2013 (39) | Low pressure RF sputtering on dry PMMA discs | N/P | N/P | He+O_2_ (10-20% O_2_) | 2 sccm of 120 secs | N/P | 10cm | 2 min. | 30W | RF sputter (Dressler Cesar 136 RF Generator,  Advanced Energy Industries; Ft Collins, CO, USA) |
| Ballout H et al. 2018 (48) | Plasma jet; DBD plasma | CAP I N/P CAP II 0.3 kHz | CAP I N/P CAP II10 kV | Ar | CAP I: 4.3 sL min ^-1^ | N/P | CAP I 3 mm  CAP II 2 mm | CAP I 60sec CAP II 60 sec | CAP II: 450 mW | CAP I: neoplasm tools GmbH, Greifswald,  Germany CAP II: Cinogy, Dudenstadt, Germany |
| Cao Y et al. 2011 (33) | Plasma jet | 10 kHz | 8 kV | He+2% O_2_ | 1 L/min | N/P | 10mm | 5,10 and 15 min. | N/P | Home-made device |
| Chang Y et al. 2016 (32) | Planar DBD plasma | 2.6 kHz | N/P | Ambient air | N/P | N/P | N/P | 0 min. (control group) 1 min 2 min 3 min 5 min 10 min 15 min | 1.1 W | Home-made device |
| Charoux C et al. 2020 (44) | Plasma jet | 20 kHz | 30 kV | Ambient air | N/P | N/P | N/P | 30 min | N/P | Diener electronic GmbH & Co.  KG, Ebhausen, Germany |
| Delben JA et al. 2014 (42) | Plasma jet | 0.05/0.060 kHz) | 220 V | Ar | 5  slm | 30.67 ± 0.58°C at 3mm and 29.83 ± 0.29°C at 10mm | 3-10mm | 15,30,60,90,120 sec | 8 W | LTP jet (Kinpen) developed by the Leibniz Institute for Plasma  Science and Technology (Greifswald, Germany) |
| Du T et al. 2012 (56) | Plasma jet | 8 kHz | 8 kV | He+O_2_ | 1:0.01 L/min | N/P | N/P | 5 min.  10 min.  15 min. | N/P | Model RC-1 |
| Habib M et al. 2014 (57) | Brush shaped plasma jet | N/P | N/P | Ar+  10% O_2_ | 500-3500 sccm | 30°C-65°C | N/P | 2 min. | 5W-15W | Home-made device |
| Hirano Y et al. 2019 (36) | Plasma jet | 0,2 kHz | 100 V | Ambient air | 1.0 L/min. | N/P | 1, 3, and 5 mm | 0,1,3,5,7 min. | N/P | Home-made device |
| Huefner A et al. 2017 (49) | Plasma jet | N/P | N/P | Ar and  Ar + 1% O_2_ | 5 slm | N/P | 3 mm | 6 min 12 min. | N/P | kINPen08, INP, Greifswald, Germany |
| Jablonowski L et al. 2013 (37) | Plasma jet | N/P | N/P | Ar | 5.0L/min. | 42 °C | 2 mm | 3 min. | 8W | kINPen08, INP, Greifswald,Germany |
| Kaya B et al. 2014 (52) | Plasma jet | 10 kHZ | 16 kV | He+O_2_ | 0.2 L/min and 5 L/min | N/P | 1 mm | 5 min | N/P | Home-made device |
| Kerlikowski A et al. 2020 (61) | Plasma jet | N/P | N/P | Ar + 1 % O_2_ | 5 slm | N/P | 1 to 2 mm | 6 min 12 min | 8W | kINPen08, INP, Greifswald, Germany |
| Kumar P et al. 2023 (58) | Plasma jet | N/P | 5 kV | He | 2,4 L/min. | N/P | N/P | 2 min  5 min.  10 min. | N/P | Home-made device |
| Leite P et al. 2021 (41) | Plasma  jet | 32.0 kHz | 13 kV | 99.5% He | N/P | <40°C | 15mm | 5 min. | 1 W | Home-made device |
| Li Y et al. 2015 (50) | Plasma jet | N/P | N/P | Ar +  2% O_2_ | 5 L/min | 30.1 °C and  27.6 °C | 10 mm | 3, 6, 9, and 12 min | N/P | Home-made device |
| Liu T et al. 2017 (45) | Brush Plasma jet | N/P | 0.8 kV | Ar  1% O_2_ | O_2_=30 sccm  Ar=3000 sccm | N/P | 5mm | 2 min. | 5W to 15W | Home-made device |
| Maisch T et al. 2012 (40) | SMDP | 1 kHz | 9 kV | Ambient air | N/P | N/P | N/P | 20, 40, 60, 120 sec. 5,6,7,8,9, 10 min. | N/P | Home-made device |
| Matthes R et al. 2015 (43) | Volume DBD | 40 kHz | 10 kV | Ar and  Ar+ 1% O_2_ | 50 sccm | N/P | 15mm | 1 ,5, 10 min | N/P | Plasma Science and Technology e.V. (INP Greifswald,  Germany) |
| Pan J et al. 2013 (1) | Plasma jet | N/P | N/P | Ar+ 2% O_2_ | 5 L/min. | 25°C to 31°C | 5 mm | 2,4,6,8 and 10 minutes | N/P | Home-made device |
| Schaudinn C et al. 2013 (60) | Plasma jet | 1.5 kHZ | 6.5 kV | He+1% O_2_ | 1 L/min. | N/P | N/P | 30 min (3x10 min with  2 min. pauses in between) | below 0.5W | Home-made device |
| Theinkom F et al. 2019 (35) | SMDP | 4.0 kHz | 3.5 kV_PP_ | Ambient air | N/P | 21°C | 20mm | 1, 3, 5 and 10 min | 0.5 to 1W | Home-made device |
| Wang R et al. 2011 (51) | Plasma jet | N/P | 400-600V | Ar + 2% O_2_ | 5 slm | 40°C | 5mm | 2,4,6 and 8 min. | N/P | Home-made device |
| Wen Y et al. 2022 (53) | Liquid direct cold plasma (plasma jet in liquid) | 100 kHz | 125 V | N/P | N/P | between 40 °C  and 70 °C | N/P | 1,3,5 and 10 min. | N/P | home-made device |
| Yoo E et al. 2020 (46) | Plasma jet | N/P | 17 kV | N_2_ | 3 L/min | N/P | N/P | 30, 60, or 120 s | N/P | Home-made device |
| Zhou X et al. 2010 (34) | Plasma jet | 8 kHZ | 8 kV | He+O_2_ (1%) | 2 L/min | N/P | N/P | 4,8 and 12 min. | N/P | Home-made device |
| Zhou X et al. 2016 (59) | Plasma jet (bubbling or not in H_2_O_2_ solution) | 8 kHz | 8 kV | He + H_2_O_2_ solution vapors | 2 l/min | N/P | N/P | 0,1,2,4 min. | N/P | Home-made device |
| Zhu M et al. 2023 (38) | Cold plasma-loaded microbubbles  (PMBs) | N/P | N/P | N/P | N/P | 40°C | N/P | 5 min. | N/P | Home- made device |
